# How task‐sharing in abortion care became the norm in Sweden: A case study of historic and current determinants and events

**DOI:** 10.1002/ijgo.13003

**Published:** 2020-07-31

**Authors:** Margit Endler, Amanda Cleeve, Ingrid Sääv, Kristina Gemzell‐Danielsson

**Affiliations:** ^1^ Department of Women's and Children's Health Karolinska Institutet Stockholm Sweden; ^2^ Women's Health Research Unit University of Cape Town Cape Town South Africa

**Keywords:** Abortion care, Health policy, Safe abortion, Sweden, Task‐sharing, Task‐shifting, Unsafe abortion

## Abstract

We performed a country case study using thematic analysis of interviews and existing grey and published literature to identify facilitators and barriers to the implementation of midwife‐provided abortion care in Sweden. Identified facilitating factors were: (1) the historical role and high status of Swedish midwives; (2) Swedish research and development of medical abortion that enabled an enlarged clinical role for midwives; (3) collaborations between individual clinicians and researchers within the professional associations, and the autonomy of clinical units to implement changes in clinical practice; (4) a historic precedent of changes in abortion policy occurring without prior official or legal sanction; (5) a context of liberal abortion laws, secularity, gender equality, public support for abortion, trust in public institutions; and (6) an increasing global interest in task‐shifting to increase access and reduce costs. Identified barriers/risks were: (1) the lack of systems for monitoring and evaluation; and (2) a loss of physician competence in abortion care.

## Abstract 2

The transition to task‐sharing abortion care in Sweden was facilitated by the role of midwives, clinical abortion research, individual autonomy, flexible legal and policy interpretation, and secular societal values.

## 1 Introduction

Task‐sharing or shifting within abortion care from specialist physicians to nonspecialist physicians or nonphysician staff has, in some contexts, been associated with a reduction in abortion‐related deaths.^1^, ^2^, ^3^, ^4^ In other contexts it has been used to increase access, cut costs, and increase continuity of care without decreasing quality of care.^5^, ^6^, ^7^

The aim of the present review was to understand how the Swedish model of task‐sharing in abortion care developed. We specifically wanted to know which factors facilitated or hindered its implementation and what impact the new model has had on access, quality, and perceptions of care.

### 1.1 The Swedish healthcare system

Healthcare policy change in Sweden can be effected at the state or regional level. The role of the Swedish government and parliament is to establish the overall principles and guidelines of healthcare provision through laws and regulations, with technical support from public health institutions and the professional organizations. The 21 regional administrations are responsible for the provision of healthcare within their constituency and autonomously perform quality controls of the care provided.^8^ The 20 regional and 40 subregional hospitals can further independently decide how to organize healthcare service delivery as long as they adhere to the mandate accorded to them by the regional administration. According to the Swedish abortion law, abortion care must be initiated either from a hospital or from a clinic approved for this purpose by the National Board of Health and Welfare (NBoHW).^9^ Abortions are offered at approximately 130 gynecological departments and specialized public or private gynecological clinics but are not offered at primary‐care level.^10^ The task‐shifting reform within abortion care in Sweden has primarily occurred at gynecological departments in regional or subregional hospitals.

### 1.2 The Swedish model of task‐sharing in abortion care

The current Swedish abortion legislature from 1975 (SFS 1974:595) allows for abortion until gestational week 18 weeks 0 days at the woman's own request.^9^ In 2017, 37 000 induced abortions were performed (20 per 1000 women aged 15–44 years).^11^ A high percentage of abortions were performed using medical methods (93%) and before the ninth week of pregnancy (84%).^11^

In 2007, the Swedish Society of Obstetrics and Gynecology (SFOG) took on a mandate, suggested by NBoHW, to develop a formal training program for abortion care certification of midwives. The first midwife‐operated abortion care unit opened in 2009, in 2012 there were nine operating units, and today there are an estimated 50 operating units out of approximately 130 clinics offering abortion care. The first formal course for midwives in abortion care and ultrasound was created by the SFOG Reference Group for Reproductive Health (FARG) in collaboration with the WHO Collaborating Centre for Research and Research Training in Human Reproduction at Karolinska Institutet (WHO Collaborating Centre). It was first held in 2013 by SFOG and the Swedish Association of Midwives (SBF) jointly.

The requirements and clinical applications of SFOG‐certified abortion care provided by midwives in Sweden are summarized in Table 11.

**Table 1:** Requirements for SFOG‐certification in abortion care and clinical applications of midwife‐provided care in Sweden

Training in abortion care^10^	Coursework	Theoretical course and exam Ultrasound course
	Clinical practice	Approval by clinical supervisor after solid clinical practice
	Documentation	Logbook of 20 ultrasounds of early pregnancy approved by course examiner
Provision of abortion care	Prerequisites	Pregnancy at <9 wk of gestation No known medical condition that complicates the abortion procedure Clinical unit has immediate access to a physician if required/requested Physician retains medical responsibility and supervision for the abortion
	First visit	Eligibility screening, information, ultrasound, medication, instructions for the procedure, contraceptive counseling and follow‐up from same midwife
	Abortion	Mifepristone taken in clinic at first visit, misoprostol taken at home, surgical abortions are performed by physicians
	Follow‐up	Self‐assessment of clinical symptoms and a low‐sensitivity pregnancy test 3 wk after the abortion

## 2 Materials and Methods

### 2.1 Data collection

We performed a country‐specific case study of task‐shifting in abortion care in Sweden. Data for the case study were assembled in two parts. The first part consisted of a literature review and the second part was a series of six in‐depth interviews with key informants.

#### 2.1.1 Literature review

In Sweden many official documents are accessible to the public as online material. The NBoHW, the Legal Database of the Office of Government (Regeringskansliets Rättsdatabaser), the Swedish Agency for Health Technology Assessment and Assessment of Social Services (SBU), The State Public Reports (SOU), and Statistics Sweden (SCB) are all public institutions that, among other things, provide statistics or analysis related to abortion incidence, abortion legislature, and abortion policy. We searched these online archives for government and nongovernmental organization reports, literature on program strategy and program outcomes, process measures, and/or monitoring and evaluation plans relevant to the development of policies and practices of nonphysician provision of abortion care. We searched PubMed for studies reporting on task‐shifting abortion care in Sweden. We performed a Google search to identify nonscientific reports, journalistic reporting, or opinion pieces related to task‐shifting abortion care in Sweden.

We organized the content of the identified documents according to the following themes: (1) the present and historic role of midwives in healthcare service delivery in general and in abortion care; (2) the institutional processes that paved the way for midwife‐provided abortion care; (3) quantitative and qualitative measures of attitudes to abortion; (4) the historic prevalence of unsafe abortion and consequences in morbidity and mortality; and (5) the history of abortion legislature, access, and practice in Sweden. The content of the assembled documentation was then synthesized in a literature review.

#### 2.1.2 Interviews

We performed individual in‐depth interviews with six key informants who played an important role in the development of midwifery‐led abortion care and were involved in implementing task‐sharing. The informants were four registered nurse‐midwives and two medical doctors with one or more current or past leadership positions in SFOG, SBF, NBoHW, the Swedish Association for Sexuality Education (RFSU), and/or the WHO Collaborating Centre at Karolinska Institutet, who performed the interviews primarily as representatives of these current or past held positions.

The informants were informed about the study and invited to participate by email. They provided consent for the content of their responses to be analyzed in terms of the aim of the study and to be quoted. The interviews were conducted in a private space with one of the researchers (ME, KG‐D, IS), or over the phone at a time chosen by the interviewee. Interviews were not recorded; however, the interviewer took detailed notes of responses in real time. Member checks were conducted by allowing each interviewee to review the transcript and make clarifications or amendments.

The interview guide was created by the Department of Reproductive Health and Research (RHR) at the WHO and consisted of open‐ended questions covering seven areas related to the policy: (1) a description of the policy in practice; (2) strategies used for implementation; (3) coalitions or collaborations involved in implementation; (4) the relationship between guidelines and practice; (5) resources demands and opportunity costs; (6) healthcare workers’ and women's experiences; and (7) monitoring/evaluation and research needs.

### 2.2 Final data analysis

The text of the literature review and the notes from each interview were read through several times to identify content relating to barriers to and facilitators in the implementation of the task‐sharing policy. We then collated the relevant content from both these sources of data into themes. A theme was defined as an aspect or idea, presented once or repeatedly in the material, that we determined was of potential influence in the success or failure of the implementation of the task‐sharing policy in the Swedish context. One researcher (ME) interpreted the data using qualitative content analysis. The content was analyzed on a manifest level (manifest analysis). Notes related to each theme were extracted and presented in a narrative summary.

## 3 Results

### 3.1 Overall assessment of the policy

All informants reported that task‐sharing has had a positive impact on abortion care and they believed that implementation had been successful. In support of this, a Swedish clinical study cited in the literature review found that women are very satisfied with midwife‐provided abortion care.^6^ Informants stated that the transition to midwife‐provided abortion care required resources in terms of training but that this cost was counterweighed because midwife provision had proved more cost‐efficient than physician‐provided care. The cost‐efficiency of midwife‐provided abortion care was supported by a study included in the literature review.^12^

Task‐sharing saves costs and reduces waiting time, thus also reduces gestational length at abortion and more resource‐demanding late abortions. It has led to higher quality [care] due to better continuity for the patient and a more effective abortion process, due to the fact that only one caregiver is involved.

Midwife, Karolinska University Hospital

Although some concern was expressed that the workload for midwives would increase, several interviewees stressed the benefits of task‐shifting for midwives, such as career development, increased salary, and involvement in research and education.

### 3.2 Facilitators and barriers to implementation of the task‐sharing policy

Eight main themes emerged through the data analysis. We identified six facilitators of the policy: the historic and contemporary role of the Swedish midwife; Swedish clinical research and the introduction of medical abortion; autonomy and role of the individual; flexibility of policy and legal interpretation; societal values (equality, secularity, and trust); and cost‐saving and service delivery incentives. We identified two potential barriers or opportunity costs to implementation: loss of competence among physicians and lack of monitoring and evaluation.

#### 3.2.1 The historic and contemporary role of the Swedish midwife

Midwifery has long been a highly autonomous and respected profession in Sweden. Between 1751 and 1900, maternal mortality in Sweden decreased from 900 to 230 per 100 000 women years.^13^ This decrease occurred before the advent of modern obstetric medicine and is thought to be largely due to community midwives. Community midwives in the 18th and 19th century were often the sole healthcare provider in Swedish parishes. Their role was recognized in a statement in 1751 by the Commission of Health (Sundhetskomissionen) that estimated that 400 out of the then 651 maternal deaths per year could be prevented if a midwife were present.^14^ Formal midwifery education was established in 1757 and by the mid‐1800s midwifery was a respectable occupation for women of any social background. By the late 1800s, 78% of women gave birth at home attended by a licensed midwife. Today, 98% of women in Sweden give birth in hospital, but a healthy woman without pregnancy‐ or delivery‐related complications will likely have all her prenatal care, ultrasound exams, birth assistance, and postnatal care conducted by a midwife, working independently, with access to an obstetrician only in case of complications.^15^

Interviews revealed that midwives’ strong position in society and public health institutions give them a prominent role in policy development, which facilitated the task‐sharing process. Several informants suggested that the broad role midwives already played in health service delivery, through Pap smear screening, youth sexual health counselling, screening for sexually transmitted infections, and contraceptive counselling, made an expanded role in abortion care seamless in the eyes of midwives and gynecologists, and completely natural in the eyes of women.

It [task‐shifting] was seen as a natural part of healthcare development. There was widespread acceptance from women and society, linked to a great confidence in midwives and their competence.

Midwife, RFSU

Midwives’ role in sexual and reproductive health care was first expanded in 1976 when they became certified to counsel and prescribe family planning methods and insert the copper intrauterine device. A second major shift in abortion care started with the introduction of medical abortion in the early 1990s, when midwives were increasingly delegated to perform the components of abortion care that conformed well to their professional role, such as contraceptive counselling, the administration of medication, supervision and pain relief during the procedure, and follow‐up.^16^ By the time formal midwife training was established, midwives already performed most of the subtasks of medical abortion care. The following quote illustrates the role of midwives at the time formal training of midwives was first considered:

I was around this time approached by colleagues with the question of whether midwives might be trained to perform ultrasound assessments in abortion care. These physicians had noted that midwives […] performed all aspects of the abortion care process apart from ultrasound. Midwives were also expressing frustration at having to call a doctor only for this component of the process.

MD, WHO Collaborating Centre, Karolinska Institutet and FARG

Whether the professional providing care was a physician or a midwife was said to be of low importance to women.

[…] this task shift comes very naturally to the Swedish society as a majority of gynecologists are women, healthy women are in contact with midwives for birth, contraception, and sexual and reproductive services, and frequently have no idea whether the person they meet at the abortion clinic is a female doctor or a midwife. Midwife, SBF

Women do not reflect on who provides the abortion care, they only want to get the abortion performed. Women turn to midwives for many services, youth counselling, contraception, Pap smears, and maternal health care, so meeting them also in the context of abortion care is completely natural.

Midwife, NBoHW

#### 3.2.2 Clinical research and the introduction of medical abortion

Sweden‐based research in the 1980s and 90s was instrumental in developing the current medical abortion regimens for the first and second trimester through the conception of the combined method of a progesterone receptor modulator and a prostaglandin analogue.^17^ More recent research has investigated the task‐shifting process and simplification of abortion care. For example, several studies linked to Sweden have supported the feasibility of medical home abortion and self‐assessment of abortion completion.^18^, ^19^, ^20^, ^21^, ^22^, ^23^, ^24^, ^25^, ^26^, ^27^ A clinical trial performed in Sweden concluded that early medical abortion provided by nurse‐midwives compared with physicians was equally effective, safe, and acceptable to women.^6^ A second study found that early medical abortion provided by nurse‐midwives compared with physicians was more cost‐effective.^12^

The clinical trial showing the noninferiority of midwife‐led abortion care was of pivotal importance in convincing opponents to the policy of its worth and gaining the official sanction of professional organizations.

MD, Linköping University, SFOG, and FARG

Medical abortion methods were introduced early and today abortion care has transitioned almost completely to medical and self‐administered methods.^11^ The interviews revealed that the introduction of medical methods for abortions paved the way for an expanded role for midwives in abortion care.

[…] it became clear that counselling on medical abortion required more time and was a more important part of the medical abortion. […] Thus the involvement of midwives increased as did the interest [of midwives] to participate in abortion care.

Midwife, SBF

The importance of research and the collaboration between research units and professional societies in the implementation process were also described by several interviewed informants as facilitating.

As head of the WHO Collaborating Centre and head of the Reference Group for Reproductive Health within SFOG, I had a platform from which to perform both clinical abortion care research and drive policy change. These two forces, as well as a close working relationship with SBF and RFSU were the means by which the policy of task‐shifting abortion care to midwives was implemented successfully.

MD, WHO Collaborating Centre, Karolinska Institutet and FARG

#### 3.2.3 Autonomy and the role of the individual

Swedish hospitals and clinics have high autonomy to create their own clinical guidelines and clinical organization structures, including delegations from physicians to nonphysician staff or from nurses to auxiliary nurses.

The informants consistently stated that the implementation of task‐sharing in abortion care is up to the medical director of each gynecological department. It was said that the success of implementation depended on the presence of individual enthusiasts among the staff as well as good working relationships between individual midwives and physicians.

Advocacy occurred through good examples and nothing else […]. The units that have been successful are the ones that have had passionate activists for the change in policy.

Midwife, NBoHW

The success and speed of change varies across the country and is dependent on the leader of the facility.

Midwife, RFSU

The idea of task‐sharing and researching task‐sharing originated from clinical researchers and members of SFOG. The process of developing the idea of midwife‐provided care was described as a close working relationship between a few individuals involved in clinical abortion research and care, and in leadership positions in SFOG or SBF, and often a combination of all three. Implementation of task‐sharing in abortion care was never actively supported by the government or by civil society; there has been no formal policy for expansion, and no direct financial support either from the government or other institutions.

#### 3.2.4 Flexible policy and legal interpretation

The historic review of abortion care in Sweden highlighted two important clinical transitions: the transition from illegal to legal abortions, and from physician‐ to midwife‐provided care. Both transitions occurred in practice before they were formally sanctioned.

The abortion law of 1975 gave the legal prerequisites for a radical change in abortion policy that had already occurred in practice. During the 1930s an estimated 20 000 illegal abortions were performed each year, 75 women died, and many more were severely injured.^28^ In 1974 however, a year before the more liberal abortion law came into force, the number of legal abortions had risen to 30 000. By that time, four‐fifths of abortions were performed before week 12, and no illegal abortions were recorded.^28^ What caused this reduction in illegal and unsafe abortions, within the confines of the existing restrictive law, was a consensus around a more liberal interpretation of the grounds on which abortion could be provided.

Task‐sharing in abortion care was formally and legally investigated at a time when midwives had already gradually taken over most tasks related to abortion care (except for vaginal ultrasound). An investigation from 2008 suggested that delegating abortion care to midwives was an effective way of increasing access to abortion; it also concluded that in many clinical departments most abortion‐related tasks were already being done by midwives.^29^, ^30^ The board of SFOG finally assigned the task of developing a curriculum for midwife certification in abortion care to FARG within SFOG, which then went on to develop the current training curriculum together with the WHO Center at Karolinska Institutet.

The component of the abortion law, which states that a person with a medical degree must be responsible for the abortion procedure, has been questioned by the Swedish Society of Health Professionals (Vårdförbundet), SBF, RFSU, and some political parties. A formal motion to move the medical responsibility from physicians to midwives was put forward to parliament by the Green Party (Miljöpartiet) in 2010 and denied.^31^ The response to the motion again concluded that most abortion‐related tasks were already being done by midwives within the confines of the law. As the existing law was flexible enough to allow almost complete task‐shifting to midwives, it was decided that physicians could retain overall responsibility and supervision of abortion care to not risk reducing the quality of care.

#### 3.2.5 Societal values: equality, secularity, trust

According to a 2018 Statista poll of perceptions of abortion among countries in Europe, at 3% Sweden has the lowest proportion of people who think that abortion should be illegal. Interviews with informants confirmed the notion of abortion being viewed as a natural part of sexual and reproductive health care.

Abortion is a natural part of women's need and essential to sexual and reproductive health care, just like contraceptives, pregnancies, and deliveries.

Midwife, RFSU

During the late 1960s and early 1970s several major societal changes occurred in Sweden that probably paved the way for the new liberal abortion law, a reduction of stigma, and the expanded role of midwives. The fact that Swedish women travelled to Poland to circumvent Sweden's restrictive abortion law became widely known in 1964 after massive media attention surrounding a conference on Reproductive Health where matters were discussed. Many women from all strata of society subsequently testified in the media to the arbitrariness, humiliation, and sexual assaults they had experienced seeking permission for abortion in Sweden. This pressured the government to form the 1965 parliamentary committee tasked with formulating a new abortion law. This period was further characterized by protests in the labor movement, the women's reform movement, and the student body voicing demands to increase equality and break down what were perceived as dehumanizing structures in society and the workplace.^32^ The women's movement and the liberalization of established social and moral attitudes to sex reached maximum force in the late 1960s. Several reforms giving women equal rights in the home and the workplace followed, which allowed for a mass influx of women to the formal workforce. The concepts of alternative family structures, contraception, free sex, and abortion became much less stigmatized. On a political level the Social Democratic government promoted the concept of the “Folk Home” which rested on the three pedestals of economic growth providing for generous spending on social welfare, high trust in government and, importantly, the promotion of individual self‐fulfillment by freeing people from restrictive social structures and norms.^32^

Together with Finland and Iceland, Sweden in practice does not allow conscientious objection to abortion provision. A study of the implications of this legal framework indicated that disallowing conscientious objection facilitates good access to reproductive healthcare services by reducing barriers and delays to care and entails no negative impacts on providers.^33^ According to studies that have evaluated perceptions of abortion care among Swedish providers, both gynecologists and midwives in general support the existing abortion legislature and do not hesitate to take part in abortion care even though they sometimes experience complex and difficult situations providing this care. The character of the work is experienced as contradictory but also gratifying.^16^, ^34^

Informants consistently answered that there have been no formal advocacy campaigns, no formal dissemination of information to civil society, and no media coverage of the new policy. To the question: *“*Has there been any backlash or negative response? From whom? And what has been done to mitigate this?”, most informants simply answered “No.”

There has been no need to mitigate any negative response. […] There has been no media coverage, possibly because there is nothing radical about this change.

Midwife, NBoHW

#### 3.2.6 Cost‐saving and service delivery incentives

Several informants described trying to expand the role of midwives in abortion care during the late 1990s and early 2000s and being met with disinterest, as exhibited by the following quotes.

In 2002, I together with a colleague published a paper in the Swedish Medical Society journal [Läkartidningen] where task‐shifting to midwives in medical abortion was suggested as a step to demedicalize abortion care, reduce stigma, and increase access to care. The article was met by silence.

Midwife, SBF

When I was a midwife in a gynecological ward taking care of women undergoing second trimester abortion I proposed a written plan [to the head of department] to create a midwife‐led abortion care unit […] but the plan was shelved.

Midwife, NBoHW

Interviews further showed that, starting in 2008, an increasing cost‐consciousness, reports of long and unequal waiting times for abortion, as well as an interest in task‐shifting within health care on a global level catalyzed the development of formal midwife training in abortion care in Sweden.

The WHO […] encouraged research surrounding task‐shifting to nonphysician personnel. Simultaneously in Sweden long waiting times to abortion were noted. Together these factors created a new focus at the WHO Collaborating Centre to investigate task‐shifting abortion care to midwives.

[…]

Since home abortion was cost saving, the practice of offering home abortion as an option to women was very quickly implemented in the whole country. Since a subsequent analysis showed that midwife‐performed abortion was also cheaper, this catalyzed the implementation also of the task‐shifting policy.

MD, WHO Collaborating Centre, Karolinska Institutet and FARG

#### 3.2.7 Loss of competence among physicians

Several informants described that there was initial concern on the part of SFOG that junior doctors would lose competence in the area of abortion care and that this might become a cost to the program over time. They stressed the importance of abortion care remaining part of the training of gynecologists to avoid a loss of competence and interest in the eyes of doctors.

There was enthusiasm mixed with reluctance […]. The main worry came from physicians that the policy would have a negative impact on the training of residents and that they would get less exposure and training in abortion care.

MD, Linköping University, SFOG, FARG

A general trend in the healthcare sector in Sweden is toward specialization of care, often to the detriment of basic competence.

MD, WHO Collaborating Centre, Karolinska Institutet and FARG

#### 3.2.8 Lack of monitoring and evaluation

Interviews showed that the quality or effectiveness of the task‐sharing policy have not been assessed at a national level. Most informants assumed that NBoHW was responsible; however, according to the literature review no institution has been given the mandate to evaluate the policy and no formal systems are in place to perform this evaluation.

It is not clear who is responsible for ongoing monitoring and evaluation of the policy, it is up to each regional council to make sure that the health care in their region is built on evidence and good practice. The National Board of Health and Welfare safeguards health, welfare, and equal access to good health care through guidance by rules and knowledge support, but it is no longer a supervising or regulatory authority.

Midwife, NBoHW

Responses from informants indicated that while SFOG successfully certifies midwives in abortion care, demand for the training exceeds supply. Although it has not been officially criticized nor confirmed, a couple of informants said that the high demand of trained midwives may have led to training outside of the formal system.

Local training instead of SFOG certification has occurred and is strongly discouraged by SFOG.

MD, Linköping University, SFOG, and FARG

Several interviewed informants mentioned risks linked to an implementation without quality controls.

[I feel] concern at the idea of maximizing task‐shifting as a way to cut costs rather than to improve quality. Abortion care must always be centered on the interests of the woman and not primarily cost‐efficiency.

MD, WHO Collaborating Centre, Karolinska Institutet and FARG

## 4 Discussion

The present case study identified six facilitating factors to the task‐sharing reform in abortion care in Sweden and two potential opportunity costs. We assessed that task‐sharing between physicians and midwives was a gradual and informal process, which was implemented without significant opposition owing to a combined effect of historical, cultural, individual, and structural factors.

### 4.1 Interpretation

Our study shows that midwives have played a pivotal historic role in improving maternal health in Sweden, which may have facilitated their transition into abortion care. The consensus among interviewed informants that women perceived the policy as completely natural suggests that there were no barriers, from the public's perspective, to midwife‐provided abortion care. Previous multicountry research on task‐shifting in abortion care has shown that successful task‐shifting is influenced by providers’ willingness to provide care and their perceptions of their professional roles. In general, however, women's preferences seem to be guided more by trust, privacy, and ease of access to care than by the category of medical staff providing the care.^35^

We found that the introduction of medical abortion into clinical abortion practice in Sweden occurred early through research and that this paved the way for an enlarged role for midwives in abortion care. Research supporting the safety, effectiveness, and cost‐efficiency of midwife‐provided care was later important in creating a consensus among providers in favor of the policy. A study from the USA supports the role of research as a driving force behind successful policy reform in the area of task‐shifting in abortion care.^36^

We found that the role of the individual and collaborations between individuals involved in both clinical research, clinical practice, and the professional organizations were key in developing the idea, the scientific foundation, and the development of formal midwife‐led abortion care units in Sweden. Analogous to this we found two historic precedents of “practice before policy” in the area of abortion care in Sweden. Firstly, the transition from mostly illegally performed abortions to exclusively legally performed abortions occurred in Sweden before the law was liberalized in 1975, most probably as a result of changing attitudes toward abortion among the media, public, and ultimately the medical profession that allowed for a more liberal interpretation of the existing law. Secondly, midwives took over most abortion‐related tasks during the years preceding the institutional and legal investigation into task‐shifting abortion care. These findings illustrate the flexibility and pragmatism of the Swedish healthcare system. Motivated individuals within clinical units and clinical units within the health system have sufficient autonomy to test changes in clinical practice that are perceived as beneficial to health service delivery without formal legal or regulatory reform. Task‐sharing in abortion care was therefore not a policy that was pushed upon clinical departments and their patients, but one that developed based on the needs of these units and their patients.

Our study indicates that earlier initiatives at policy reform, taken by midwives, were unsuccessful. This may suggest that the senior gynecologists who initiated the successful reform had greater leverage, but it is also probable that their initiative occurred at a time when more research existed in support of the reform, task‐shifting had moved onto the global agenda, and service delivery concerns related to abortion were increasing. The implementation of task‐sharing in abortion care also occurred in a context of a liberal and supportive abortion law, high public support for free access to abortion, low religiosity, high gender equality, and high trust in public institutions. All these factors may have facilitated the transition to midwife‐provided abortion care.

Some of our informants expressed concern that task‐shifting in abortion care could lead to a loss of competence and interest in abortion care among physicians, particularly if abortion care was moved solely to the domain of midwives. Research supports that medical abortion care provided by midwives and other nonphysician medical staff is noninferior to physician‐provided care.^37^ The Swedish model of task‐sharing is, however, an example of resource‐efficient teamwork between midwives and gynecologists, tiering care according to the clinical needs of the woman. We argue that abortion care should remain a collaborative effort between physicians and midwives or nurses to give questions of abortion care maximum leverage on the global research and policy agenda.

There is no official record of the number of units offering midwife‐provided abortion care in Sweden and the policy has not been formally evaluated. The lack of systems for monitoring and evaluation may be problematic for the continuing success and quality of the program. Cost‐saving incentives could jeopardize the quality of care if the process is left unchecked. Furthermore, since the training of midwives is not a formal national policy and receives no government funding, there is no plan or source of funds for expansion. The fact that the task‐sharing reform was successfully implemented without formal monitoring and evaluation may be testament both to the feasibility of decentralized reform in Sweden and to the vulnerability of the policy, which does not yet have the institutionalized support and funding that would make it self‐sustainable.

### 4.2 Strengths and limitations

Task‐sharing in abortion care in Sweden occurred upon the initiative of individual clinical departments and has expanded without systematic controls, monitoring, and evaluation. This weakens the ability of this study to systematically identify facilitators and barriers to implementation. Some clinical units may have chosen not to implement the policy because of barriers that would not have been captured in this review.

A strength of this study is the existing extensive background data owing to the easy access to current and historical public policy and legal documents in Sweden, as well as the long tradition of keeping registries on birth and death rates, abortion statistics, and causes of mortality. Furthermore, the process of task‐sharing in abortion care was driven by a small number of individuals, many of whom are captured in this review. Interviews were not audio recorded but transcribed in note form and quotes verified retrospectively. One informant is also a coauthor of the article, but she did not take part in the data analysis.

In conclusion, task‐shifting abortion care to midwives in Sweden was neither a regulatory nor a legal reform but occurred at facility level within the existing health and legal framework upon the initiative of clinicians and researchers within the medical professional societies and research units. Although the process was facilitated by factors specific to the Swedish context, this model for policy change may be applicable to other settings.

## Author Contributions

All authors contributed to the study design. ME, KG‐D, and IS performed the interviews. ME performed the literature review and wrote the first draft of the article. All authors reviewed the article and approved the final manuscript.

## Conflicts of Interest

The authors have no conflicts of interest to declare.
